# *Mucuna pruriens* cannot develop phytoremediation of tebuthiuron in agricultural soil with vinasse: a morphometrical and ecotoxicological analysis

**DOI:** 10.3389/fbioe.2023.1156751

**Published:** 2023-05-04

**Authors:** Yanca Araujo Frias, Edivaldo Wilson Lima, Munick Beato Aragão, Laura Silva Nantes, Bruno Rafael Almeida Moreira, Victor Hugo Cruz, Rafael Simões Tomaz, Paulo Renato Matos Lopes

**Affiliations:** Department of Plant Production, College of Agricultural and Technological Sciences, São Paulo State University (Unesp), Dracena, Brazil

**Keywords:** bioremediation, organic compound, herbicide, green manure, ecotoxicology

## Abstract

Pesticides offer stakeholders cost-effective solutions to control weeds. Nevertheless, such active compounds can manifest as severe environmental pollutants when escaping from agroecosystems into surrounding natural ecosystems, driving the need to remediate them. We, hence, analyzed whether *Mucuna pruriens* can develop a potential phytoremediator for treating tebuthiuron (TBT) in soil with vinasse. We exposed *M. pruriens* to microenvironments containing tebuthiuron at 0.5, 1, 1.5, and 2 (standard dose) L ha^−1^ and vinasse at 75, 150 (industrial recommendation), and 300 m^3^·ha^−1^. Experimental units without organic compounds represented controls. We assessed *M. pruriens* for morphometrical features, such as plant height and stem diameter and shoot/root dry mass, over approximately 60 days. We obtained evidence for *M. pruriens* not effectively removing tebuthiuron from the terrestrial medium. Such a pesticide developed phytotoxicity, significantly limiting its germination and growth. The higher the dose, the more negatively the tebuthiuron impacted the plant. In addition, introducing vinasse into the system, irrespective of volume, intensified the damage to photosynthetic and non-photosynthetic structures. Equally important, its antagonist action further decreased the production and accumulation of biomass. As *M. pruriens* could not effectively extract tebuthiuron from the soil, it could allow neither *Crotalaria juncea* nor *Lactuca sativa* to grow on synthetic media containing residual pesticide. An atypical performance of such testing (tebuthiuron-sensitive) organisms over independent ecotoxicological bioassays validated inefficient phytoremediation. Hence, *M. pruriens* could not offer a functional remediative option to treat environmental pollution by tebuthiuron in agroecosystems where vinasse occurs, such as sugarcane-producing areas. Although *M. pruriens* considered a tebuthiuron phytoremediator as cited in the literature, satisfactory results did not occur in our research due to high concentrations of vinasse in the soil. Therefore, this information requires more specific studies about the influence of high concentrations of organic matter on *M. pruriens* productivity and phytoremediation performance.

## 1 Introduction

Full-scale producers frequently rely on chemicals, such as pesticides and fertilizers, to enhance the productivity and quality of commercially significant crops for food, energy, and fiber (Silva et al., 2019). For instance, systematically applying herbicides on commercial fields to control weeds can protect them against interspecifically competing over resources, such as physical space, radiant energy, and nutritive minerals (Silva et al., 2019). As a result, it optimizes their cost-effectiveness and profitability. Nevertheless, an overreliance on spraying such compounds or their unsuitable introduction into agroecosystems can represent a source of hazardous pollution to society and the environment, as they can drift from the objective area and move into surrounding natural ecosystems (Silva et al., 2019).

Stakeholders in Brazil’s sugar-energy sector generally base their weed management and control programs on tebuthiuron (TBT). Such an active compound represents one of the most significant phenylureas for application in intensive agricultural production systems. Tebuthiuron acts as a systemic, broad-spectrum photosystem II (PS II) inhibiting agent (Mendes et al., 2021). Hence, it can suppress broad-leaf (e.g., *Commelina benghalensis*) and grassy individuals (e.g., *Urochloa decumbens*, *Digitaria horizontalis*, and *Panicum maximum*) by disrupting their photosynthetic activity (Mendes et al., 2021).

Nevertheless, physicochemical features, such as log K_OW_ (1.8) and water-solubility (2,500 mg·L^−1^ at 20°C), facilitate its persistence in soils or movement from them to aquatic ecosystems via leaching and run-off (Faria et al., 2018). Thus, TBT can manifest as an organic pollutant of emerging concern to the surface and underground waters. Most severely, it can bioaccumulate through food webs, negatively impacting non-target organisms, such as pollinators, invertebrates, and humans (Faria et al., 2018), driving the need to address its remediation (Mercurio et al., 2015; Mercurio et al., 2016).

One of the most appealing biological strategies to address pressing environmental issues concerning the occurrence of residual TBT in agroecosystems and the surroundings is microbial bioremediation (Mercurio et al., 2015; Mercurio et al., 2016). For instance, Lima et al. (2022) selected potential TBT-degrading rhizospheric microorganisms from sugarcane-producing areas. Hence, the authors developed a bacterial pool capable of catabolizing the previous herbicide into carbon dioxide (CO_2_). Nevertheless, microbial metabolism depended on strict experimental conditions, such as purity of the analyte, temperature, luminosity, and sterility of the medium, to perform the transformation, limiting its reproduction on an industrial scale. Nantes et al. (2022) confirmed the challenging scalability of microorganisms in treating TBT in microenvironments with and without thiamethoxam. Nevertheless, the authors stressed the possibility of integrating them with crops for biocatalytic phytoremediative processes (Wei et al., 2021; Lopes et al., 2022).

Ferreira et al. (2021) contributed to advancing the field’s prominence in biologically treating TBT in agricultural soils by selecting functional phytoremediator organisms. Their models, namely, *Cajanus cajan*, *Canavalia ensiformis*, *Mucuna pruriens*, and *Pennisetum glaucum*, effectively removed the herbicide at 2 L·ha^−1^ from the medium, allowing the successful subsequent production of *C. juncea* and *L. sativa* under its residual. Equally significant, the authors established a path for re-utilizing vinasse as a source of organic carbon for vegetable growth and development, improving the environmental aspect of phytoremediation. Nevertheless, as an emerging topic, such a study may not offer stakeholders sufficient information for its development and scaling, driving the need to delve deeper into designing, analyzing, and validating environmental conditions.

Hence, leguminous plants are widely used for green manure, besides the ability to fix nitrogen and the potential as good phytoremediator since they contributes to microbial biostimulation in soil (Mendes et al., 2020; Ferreira et al., 2021; Ferreira, Lopes, 2021). These species are not only used as their agronomic interest, but also have several advantages regarding good sustainable soil management. Furthermore, this practice is linked to some of the UN’s sustainable development goals, such as: Zero Hunger (SDG 2), Responsible Consumption and Production (SDG 12), and Life on Land (SDG 15); which call for ensuring sustainable food production systems for implementing resilient agricultural practices and progressively improve land and soil quality.

Therefore, a planned broader-range research to analyze whether *M. pruriens* can develop functional phytoremediation in soil with varying analytical concentrations of TBT and volumetric applications of vinasse. Thus, this research aimed to evaluate the residual tebuthiuron impact on the technical efficacy of the biological treatment using *C. juncea* and *L. sativa* as bioindicator species in soil.

## 2 Materials and methods

### 2.1 Experimental environment and acquisition of *M. pruriens*, tebuthiuron, and vinasse

We conducted experiments in a greenhouse near 21°28′57″S and 51°31′58″W, beginning May through August 2020. The temperature and relative humidity of the air in such an environmentally controlled facility over the assessing and monitoring period was 23.4°C and 36.8%, respectively. We collected soil (Oxisol) from the Major and Minor Crop Division of the São Paulo State University (Unesp). Such material contained 89.9% sand, 7.1% clay, and 3% silt. In addition, chemically, it consisted of a slightly acidic pH (6), 2 mg·dm^−3^ P, 6.6 mmol_c_·dm^−3^·K, 27 mmol_c_·dm^−3^ Ca, 27 mmol_c_·dm^−3^·Mg, and 6 mg·dm^−3^ organic matter (Table 1). We purchased TBT (commercially available in the active composition of Combine^®^ 500 SC, Dow AgroSciences) and seeds of *M. pruriens*, *C. juncea*, and *L. sativa* from an agricultural cooperative. In addition, we acquired vinasse as a by-product of a bioethanol-fabricating line from a full-scale sugar-energy plant.

**TABLE 1:** Characterization of soil for standard textural and physicochemical properties.

Property	Unit	Analytical quantity
Phosphorus	mg dm^−3^	2
Organic matter		6
Potassium (P)	mmol_c_ dm^−3^	6.6
Calcium (Ca)		27
Magnesium (Mg)		27
Potential acidity (H^+^ plus Al)		14
Sum of bases		60.6
Cationic exchange capacity		74.6
Saturation of bases	%	81.2
Sand		89.9
Clay		7.1
Silt		3
Potential of hydrogen (pH)		6

### 2.2 Application of tebuthiuron and vinasse in soil

In Brazil, producers frequently apply 2 L of Combine^®^ (1,000 g TBT) and 150 m^3^ of vinasse per hectare on industrial fields to address a cost-effective chemical suppression of weeds and environmentally secure fertigation (Cetesb–Environmental Company of the State of São Paulo, 2015). We, hence, based our varying doses and challenging conditions (Table 2) on such reference values.

**TABLE 2:** Factorial tests for the potential phytoremediation of tebuthiuron by *M. pruriens* in soil with vinasse.

Test^a^	Factor A, tebuthiuron (L ha^−1^)	Factor B, vinasse (m^3^ ha^−1^)
1	0.5 (0.25 × standard dose)	No application ^∗∗^
2	1 (0.5 × standard dose)	
3	1.5 (0.75 × standard dose)	
4	2 (standard dose)	
5	No application^b^	
6	0.5 (0.25 × standard dose)	75 (0.5 × industrial recommendation)
7	1 (0.5 × standard dose)	
8	1.5 (0.75 × standard dose)	
9	2 (standard dose)	
10	No application^b^	
11	0.5 (0.25 × standard dose)	150 (industrial recommendation)
12	1 (0.5 × standard dose)	
13	1.5 (0.75 × standard dose)	
14	2 (standard dose)	
15	No application^b^	
16	0.5 (0.25 × standard dose)	300 (2 × industrial recommendation)
17	1 (0.5 × standard dose)	
18	1.5 (0.75 × standard dose)	
19	2 (standard dose)	
20	No application^b^	

^a^ Every testing condition comprised four replicates to reduce systematic (non-random) errors and ensure the reproducibility of the study.

^b^ Application of water to establish control.

We sieved the soil to select homogeneous particles of 2 mm. In addition, we thoroughly mixed it with vinasse by hand in individual sterile plastic trays. We transferred aliquots of 0.3 m^3^ to 3.6 L pots and then randomly arranged them in the greenhouse for chemical treatment. We, hence, applied TBT on experimental units via a backpack device (Herbicate^®^), which provides a suite of XR 11002 flat nozzles every 0.5 m over a 1 m bar for broadcast spraying. We configurated the sprayer to release such herbicide at 200 kPa and 0.65 L·min^−1^. Equally significant, we operated it at 5 km·h^−1^ and 0.75 m high from the top of the pots, as per Ferreira et al. (2021). Temperature, relative humidity of the air, and wind speed during spraying were 23.5°C, 50.9%, and 2.2 m·s^−1^, as recorded by a portable digital thermo-hygro-anemometer-luxmeter (THAL-300, Instrutherm^®^). We added water to tests without TBT and vinasse to establish controls.

### 2.3 Implementation, monitoring, and assessment of *M. pruriens* and *C. juncea*

After 7 days of spraying, we implemented *M. pruriens* in pots. We allocated three seeds per experimental unit. On the 14th day of production, we thinned two seedlings to guarantee the development of one vigorous individual. We assessed such a potential phytoremediator for standard morphometrical features, such as plant height and stem diameter and shoot/root dry mass, over approximately 60 days (Ferreira et al., 2021). After 7 days of *M. pruriens* harvesting, we introduced *C. juncea* as a bioindicator of residual TBT into the system. We distributed seven seeds per pot. After 14 days of sowing, we thinned seedlings to establish one sentinel plant per experimental unit. We investigated *C. juncea* for the same biometric markers of growth and development. We managed both species strictly as per technical recommendations for green manure. For instance, they received daily irrigation via automatic micro-sprinklers at 6 mm·h^−1^ for 1 h. In addition, we removed weeds by hand to protect them against interspecific competition over resources, such as physical space, sunlight, and nutritive minerals (Ferreira et al., 2021).

### 2.4 Ecotoxicological bioassay

We recovered 1 g of soil from experimental units at the commencement (t_0_) and after approximately 5 months (t_140_) of the successive production of *M. pruriens* and *C. juncea*. In addition, we introduced such a quantity of material into glass tubes containing 10 mL of ultra-pure water to establish analytical solutions. Next, we pipetted 2 mL to apply on sterile Petri dishes with ten seeds of *L. sativa*. Then we incubated them in a climatic chamber at 25°C ± 2°C, 60% ± 5%, and 12 h photoperiod. We monitored the growth and development of such a bioindicator of residual toxicity for 5 days (Lima et al., 2022). We quantified its percentage germination by visual counting.

### 2.5 Chromatographic quantification of residual tebuthiuron in soil

We conducted high-performance liquid chromatography (HPLC) to quantify residual TBT in the soil at t_0_ and t_140_. We programmed the isocratic HPLC-UV/Vis system to detect such an analyte in a mobile phase consisting of methanol and water (1:1, v v^−1^) at 254 nm, 30°C, and 1 mL·min^−1^ (Moreno-González et al., 2018).

### 2.6 Statistical analysis

We applied a response-surface approach to agronomic and ecotoxicological data to model the impact of (residual) tebuthiuron and vinasse on *M. pruriens*, *C. juncea*, and *L. sativa*. We designed isocurve diagrams rather than tables of numerical data to facilitate the identification and interpretation of eventual modifications in vegetable morphometrical and developmental features by prospective readers. We ran analytical protocols in software R for statistical computing and graphics (R Core Team, 2020).

## 3 Results and discussion

### 3.1 Morphometrical features of *M. pruriens* in soil with tebuthiuron and vinasse

**FIGURE 1:**
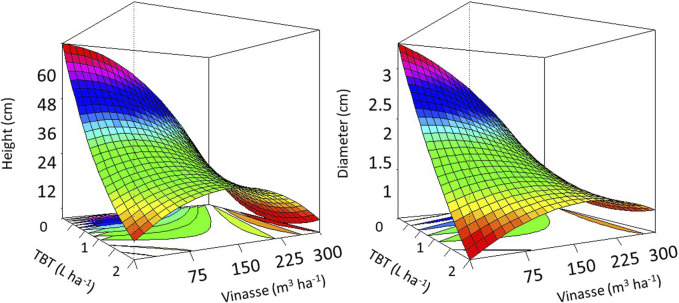
The interactive effect of tebuthiuron and vinasse on morphometrical features of *Mucuna pruriens*. Higher values occur as the frames in the response-surface diagrams modify from scarlet to fuchsia.

By analyzing the response-surface diagram (Figure 1), we can recognize the significant impact of tebuthiuron and vinasse on the stem diameter and plant height of *M. pruriens*. Such substances negatively affected both morphometrical indicators of primary growth and development.

*M. pruriens* developed the highest values of plant height and stem diameter of approximately 69.8 and 3.45 cm in reference units. Nevertheless, as we introduced TBT at the highest concentration into the system, their corresponding measures decreased to 10.4 and 0.2 cm. We identified similar downward trends for the single effect of vinasse, as its occurrence at the highest volumetric application decreased plant height and stem diameter to 22.9 and 1.3 cm, respectively. Hence, such features supported an antagonism of both substances toward the developmental performance of *M. pruriens* and its inability to overcome a harsher microenvironment (Silva et al., 2012).

**FIGURE 2:**
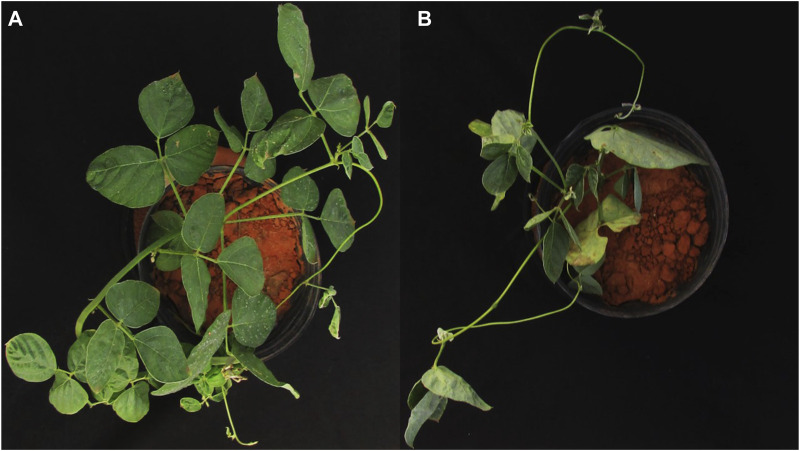
Visual aspect of *M. pruriens* in agricultural soil without **(A)** and with **(B)** tebuthiuron (2 L·ha^−1^) and vinasse (300 m^3^·ha^−1^).

The highest concentration of TBT most decreased plant height and stem diameter. Nevertheless, its interaction with vinasse at 150 m^3^·ha^−1^ allowed the *M. pruriens* to develop higher measures for such morphometric characteristics. We, hence, obtained evidence for such an industrial by-product reducing the phytotoxicity of the herbicide at a standard and environmentally acceptable volumetric application. Perhaps it acted as an organic source of energy (i.e., carbon) to the *M. pruriens*, allowing it to further develop its architectural structures in a medium where highly harmful TBT occurred at 2 L·ha^−1^. The fertilizing role of vinasse attenuated the negative impact of TBT at the highest concentration on the longitudinal and sectional planes of *M. pruriens*. Nevertheless, it could not be technically sufficient to enable such a species to develop a functional phytoremediation. Its considerably lower growth and development than usual (Figure 2) could not support an effective removal of TBT from the soil.

### 3.2 Morphometrical features of *C. juncea* in soil remaining in the production of *M. pruriens*

**FIGURE 3:**
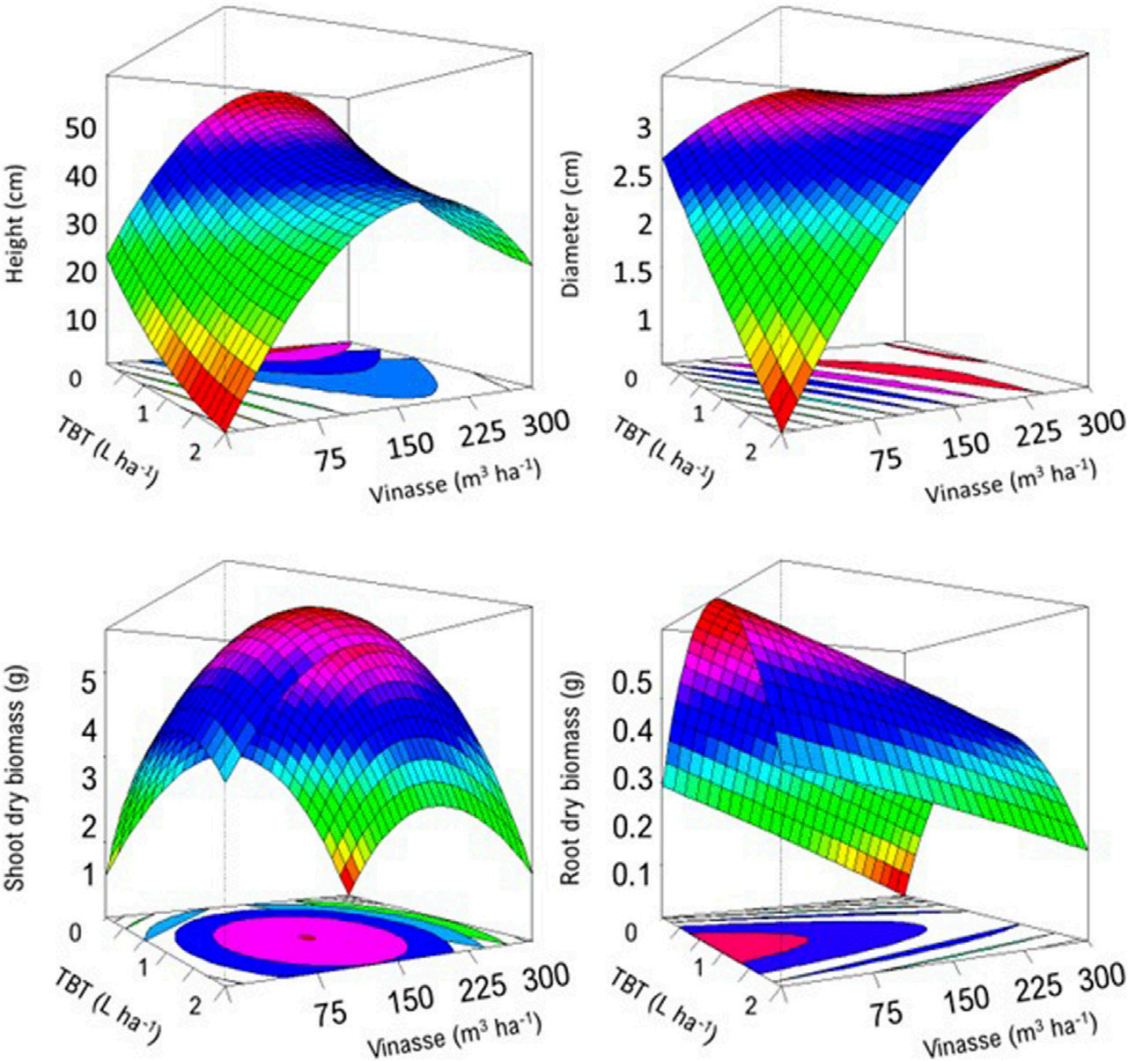
The interactive effect of residual tebuthiuron and vinasse on morphometrical features of *C. juncea*. Higher values occur as the frames in the response-surface diagrams modify from scarlet to fuchsia.

A plant capable of vigorously growing and developing stabilizer or extractor structures under a pollutant organic compound, such as TBT, is crucial to its detoxification from a substratum; otherwise, it may not offer stakeholders a suitable and scalable biodynamic option for environmental reclamation, as is the case of *M. pruriens*. As a result of the insignificant remediation performance of *M. pruriens*, the amount of pesticide remaining in the soil severely limited the subsequent production of *C. juncea* (Figure 3).

**FIGURE 4:**
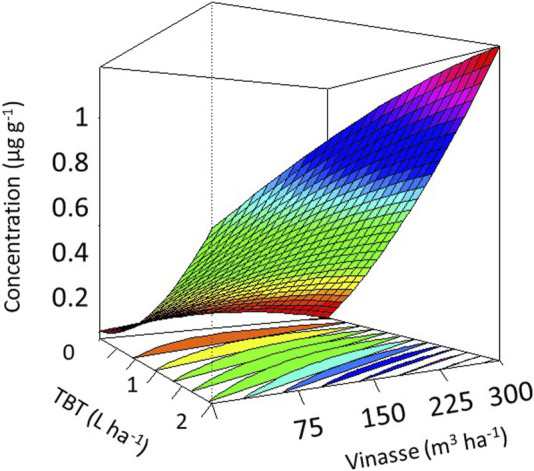
Concentration of residual tebuthiuron in soil after the successive production of *M. pruriens* and *C. juncea*. Higher values occur as the frames in the response-surface diagrams modify from scarlet to fuchsia.

The higher the analytical concentration of TBT, the less effective its removal from the soil by *M. pruriens*. Hence, 2 L·ha^−1^ developed the harshest microenvironments for *C. juncea*, significantly decreasing its plant height and stem diameter to 2.1 and 0.15 cm, respectively. We measured approximately 23.4 and 2.7 cm from reference units, supporting the persistence of phytotoxic TBT in the medium (Figure 4) and the systematic sensitiveness of *C. juncea*. Pires et al. (2008) and Ferreira et al. (2021) demonstrated the biomarking role of such a species in their independent studies on selecting potential phytoremediators to treat TBT in soil.

As a significant quantity (0.025–1.3 μg g^−1^) of residual herbicide occurred in soil remaining in the production of *M. pruriens*, it reduced the agronomic performance of the subsequent experimental cultivation. Nevertheless, introducing vinasse into the system, irrespective of the volumetric application, allowed it for additional growth and development, supporting its fertilizing role and compensatory action for the phytotoxicity of the herbicide (Souza et al., 2009). For instance, *C. juncea* developed plant height and stem diameter of 28.5 and 3.4 cm, respectively, at 2 L·ha^−1^ of TBT and 300 m^3^·ha^−1^ of vinasse. Such morphometric measures were slightly higher than the corresponding values for reference units.

**FIGURE 5:**
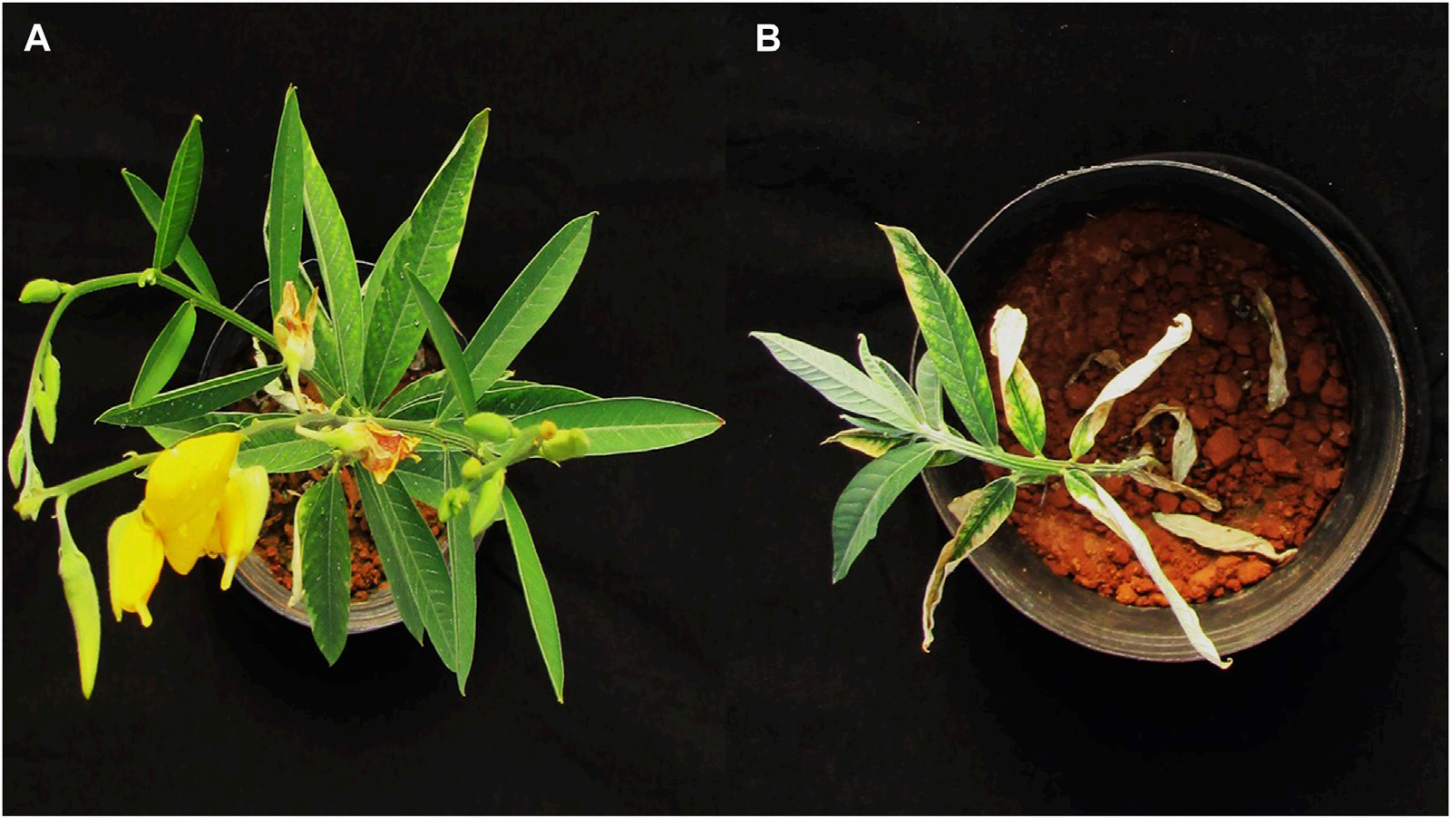
Visual aspect of *C. juncea* in agricultural soil without **(A)** and with **(B)** tebuthiuron (2 L·ha^−1^) and vinasse (300 m^3^·ha^−1^).

Comparatively, 150 m^3^·ha^−1^ outperformed 300 m^3^·ha^−1^ in *C. juncea* production. Therefore, excessive vinasse acted antagonistically on plant growth and development (Ferreira et al., 2021; Ogura et al., 2022), decreasing stem diameter of *C. juncea*. In addition, vinasse significantly reduced *C. juncea* dry mass production and accumulation (Figure 5). We identified a similar downward trend for the single effect of TBT, as *C. juncea* developed the highest quantities of dry shoots and roots at 1 L·ha^−1^. Such an outperformance supported a higher severity of 2 L·ha^−1^ over both *M. pruriens* and *C. juncea*. Mendes et al. (2020) provided evidence of the ability of TBT to reduce the productivity of dry biomass by *C. ensiformis*, *Lupinus albus*, and *C. spectabilis* by 9.45%, 11.4%, and 50.2%, respectively. Supporting our trends. Upscaling the volumetric application of vinasse from 75 to 300 m^3^·ha^−1^ increased the analytical concentration of residual TBT in the soil. Therefore, the higher the quantity of such an organic by-product in the system, the higher the availability of functional binding sites to retain such a reactive herbicide (Trevisan et al., 2016; Mendes et al., 2021), decreasing its effective removal by *M. pruriens*.

### 3.3 Ecotoxicological effects of residual tebuthiuron on germination and development of *L. sativa*

**FIGURE 6:**
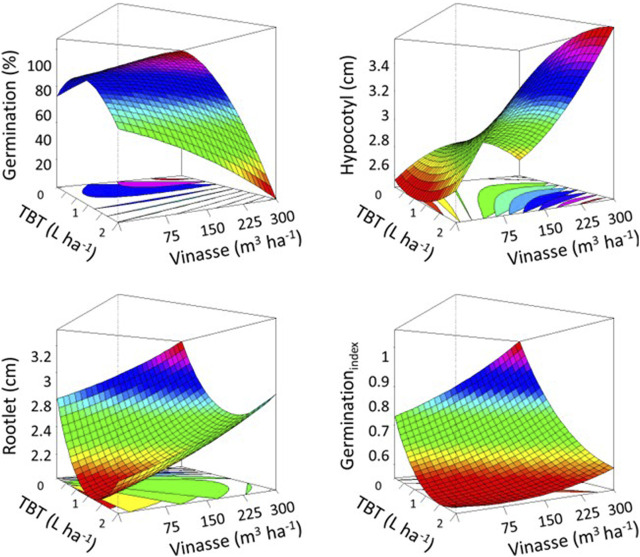
Ecotoxicological impact of residual tebuthiuron on the germinative capability and developmental performance of *L. sativa* on plates containing soil from the successive production of *M. pruriens* and *C. juncea*. Higher values occur as the frames in the response-surface diagrams modify from scarlet to fuchsia.

*L. sativa* further germinated and developed hypocotyl and rootlet in plates containing soil from reference units (Figure 6). Such a trend validated inefficient phytoremediation by *M. pruriens* and phytotoxic residual TBT. As such herbicide occurred in samples remaining in the successive production of *M. pruriens* and *C. juncea*, it decreased the germinative potential and developmental performance of the testing organism. Equally significant, it damaged and promoted atypical features in its seedlings, such as an unbalanced hypocotyl-to-rootlet ratio.

As vinasse manifested as a fertilizing and attenuating agent for the production of *M. pruriens* under phytotoxic TBT, its occurrence on Petri dishes allowed *L. sativa* to develop a higher percentage of germination. In addition, it enhanced the measures of its hypocotyl and rootlet. As a result, it either maximized the germination index or alleviated the negative impact of the herbicide on such a derivative biophysical metric, as evidenced by our response-surface charts. Such digital representations of our experimentation further supported the antagonistic effect of vinasse at increasing volumes on vegetable growth and development, as it may excessively contain harmful organic and inorganic substances for vegetable growth and development, such as mineral ions and heavy metals (Ferreira et al., 2021). In addition, it may retain TBT, intensifying the ecotoxicological potential of such an association over *L. sativa.*

### 3.4 Implications to advance the field’s prominence in developing phytoremediation of tebuthiuron

As emerging topics, studies on disruptive solutions for the bioremediation of TBT concentrate on plants (Ferreira et al., 2021) and microorganisms (Lima et al., 2022; Nantes et al., 2022). They provide significant knowledge about selecting suitable biotechnological models for treating such a pesticide in agricultural soils. Nevertheless, they are still at an early stage of development, driving the need to delve deeper into designing, analyzing, and validating experimental conditions for reliable reproduction at an industrial scale. For instance, Ferreira et al. (2021) demonstrated the ability of *C. cajan*, *P. glaucum*, *C. ensiformis*, and *M. pruriens* to extract TBT in microenvironments with and without vinasse as an additional source of carbon for the plant metabolism. *P. glaucum* and *M. pruriens* outperformed *C. cajan* and *C. ensiformis* in remediating TBT at a standard concentration of 2 L·ha^−1^. The authors optimized their approach by introducing vinasse at 150 m^3^·ha^−1^ into biosystems.

Our wider-range research could not confirm those trends in the earlier investigation by Ferreira et al. (2021) since *M. pruriens* could not develop functional phytoremediation of such an organic compound at varying concentrations at 0.5, 1, 1.5, and 2 L·ha^−1^. Even though vinasse at volumetric applications between 75 and 300 m^3^·ha^−1^ attenuated the chemical pressure by TBT over *M. pruriens*, allowing it to further growth and development, it could not be sufficient for significant detoxification. In addition, such an organic by-product retained TBT, decreasing its bioavailability for the potential remediative biosystem while favoring the persistence of its toxic residual in the soil. As a result, it reduced the germination and development of *L. sativa*. As vinasse can negatively impact the removal of TBT by *M. pruriens*, prospective stakeholders must balance its benefits (e.g., fertilizing role) and trade-offs (pesticide-binding capability) in treating such an organic pollutant of emerging concern in agroecosystems where its fertigation occurs. Equally important, they must focus on developing an alternative to *M. pruriens*, as it cannot offer them a consistent option for environmental reclamation through either phytoextraction or phytostabilization. Perhaps TBT-degrading microorganisms, such as available bacteria and fungi in the rhizosphere of sugarcane (Lima et al., 2022), enhance the remediative performance of *M. pruriens*. Hence, stakeholders must consider their integration in elaborating on and implementing biocatalytic systems for high-throughput intervention.

## 4 Conclusion

We investigated *M. pruriens* for the potential phytoremediation of tebuthiuron in soil with vinasse. This biological model negatively responded to the chemical treatment. Hence, it cannot offer an effective solution to extract the previous herbicide from the medium. Vinasse manifested as a fertilizing agent to the *M. pruriens*, which improved its growth and development in microenvironments containing the target pesticide while maintaining an environmentally sustainable feature at 150 m^3^·ha^−1^. Nevertheless, it could not be sufficient to support functional phytoremediation and subsequently significant detoxification. Furthermore, it could undesirably retain tebuthiuron through adsorption on inherent binding sites and decrease its bioavailability to *M. pruriens* while increasing its residual concentration in soil. As a result, the pesticide persisted in the system at harmful quantities to *C. juncea* and *L. sativa*, whose germination and expansion decreased and most severely caused mortality. Our fundamental and analytical insights provided knowledge to progress the field’s prominence in developing phytoremediation for biologically treating tebuthiuron in intensive ecosystems, such as sugarcane-producing areas. Stakeholders may benefit from them to avoid unnecessarily consuming resources (e.g., time, labor, and funding) in researching for and upscaling evidence-based inefficient species. Finally, high concentrations of vinasse in the soil not promoted satisfactory results on *M. pruriens* phytoremediator capacity. Therefore, this evaluation requires more specific studies to investigate deeply the influence of organic matter associated to tebuthiuron in soil for *M. pruriens* productivity and phytoremediation performance.

## Data Availability

The original contributions presented in the study are included in the article, further inquiries can be directed to the corresponding author.
